# Voxel-FPN: Multi-Scale Voxel Feature Aggregation for 3D Object Detection from LIDAR Point Clouds

**DOI:** 10.3390/s20030704

**Published:** 2020-01-28

**Authors:** Hongwu Kuang, Bei Wang, Jianping An, Ming Zhang, Zehan Zhang

**Affiliations:** Hangzhou Hikvision Digital Technology Co. Ltd., Hangzhou 310052, China; kuanghongwu@hikvision.com (H.K.); anjianping@hikvision.com (J.A.); zhangming15@hikvision.com (M.Z.); zhangzehan@hikvision.com (Z.Z.)

**Keywords:** 3D object detection, multi-scale voxel feature aggregation, LIDAR, autonomous driving

## Abstract

Object detection in point cloud data is one of the key components in computer vision systems, especially for autonomous driving applications. In this work, we present Voxel-Feature Pyramid Network, a novel one-stage 3D object detector that utilizes raw data from LIDAR sensors only. The core framework consists of an encoder network and a corresponding decoder followed by a region proposal network. Encoder extracts and fuses multi-scale voxel information in a bottom-up manner, whereas decoder fuses multiple feature maps from various scales by Feature Pyramid Network in a top-down way. Extensive experiments show that the proposed method has better performance on extracting features from point data and demonstrates its superiority over some baselines on the challenging KITTI-3D benchmark, obtaining good performance on both speed and accuracy in real-world scenarios.

## 1. Introduction

The great success of deep learning networks in 2D image detection [1,2,3,4,5,6] has accelerated the development of 3D object detection techniques. Although, provided with extra depth information from 3D point cloud, the difference of data modality between 3D point clouds and 2D RGB images makes it a big challenge in directly transplanting 2D detection techniques. Moreover, with the increase of dimensions and degrees-of-freedom, the objective of predicting exact position, size, and orientation in 3D space requires highly-demanding efforts.

In autonomous driving applications, RGB images and 3D point clouds could be simultaneously captured by camera and LIDAR sensors. Using either or both of the two modalities, researchers explore effective and reliable solutions for 3D object detection tasks. In terms of representation learning, state-of-the-art work of 3D object detection could be divided into three kinds of methodology in whole: (a) fusion-based approaches, which synchronously fuse region features from RGB images and preprocessed 3D point clouds [7,8,9]; (b) 2D-detection-driven methods, to conduct subsequent object search in 3D subspace extended from 2D bounding boxes of detection results in RGB images [10]; and (c) point-cloud-based methods, exploring the features and inner topology of points to detect 3D objects [11,12,13,14,15,16,17].

Currently, fusion-based methods require mature 2D detection frameworks to project the underlying point clouds into bird’s eye view (BEV), which might lead to certain degree of information loss. Although, for 2D-detection–driven approaches, missing objects in RGB image may cause detection failure. Meanwhile, pioneering works [18] on processing raw point clouds have been proposed and further developed to meet the growing demands for higher accuracy on 3D detection.

Recently, a new method called PointPillars [19] is proposed. This approach combines raw point cloud and voxel-based feature extraction into an efficient end-to-end network. It first organizes raw point clouds as pillars, and then applies PointNet to learn the representation of point clouds. On this basis, a standard 2D convolutional is used for the final detection, enabling efficient real-time detection. As of now, this is a state-of-the-art method. However, to achieve good detection results, this method must carefully select the pillar size [19] (the size of the voxel). In other words, researchers need to spend a lot of effort to find the right voxel size to get good detection results.

In this paper, we propose a new detection method called Voxel-FPN (Feature Pyramid Network [20]), which addresses the above challenges by integrating multi-scale voxel features with FPN. We present main workflow of the 3D detector, which can be viewed a classic encoder–decoder framework that directly operates on point clouds. Unlike existing voxel-based approaches that only utilize points from single scale via a direct forwarding route, we encode multi-scaled voxel features from point data and then aggregate these features via a bottom-up pathway. Two corresponding feature pyramid networks (FPN) are designed to decode these feature maps from various scales and associate them with final detection outputs. Experiments indicate that the proposed framework obtains a good balance between speed and accuracy for real-time applications.

Benefiting from the multi-scale Voxel representation and FPN designed in this paper, our approach has the following advantages. First, by learning multi-scale voxel features, there is no need to tune the voxel size by hand; second, multi-scale voxel features can be succinctly fused together by FPN for fast and efficient detection. With this simple structure, the proposed framework obtains a good balance between speed and accuracy for real-time applications. In summary, our contribution resides as follows.

- We propose a fast and practical 3D detection framework based on point clouds, exploiting multi-scale voxel partitioning.
- We design a VFE (Voxel Feature Encoding)-FPN network to enable early fusion of multi-scale voxel information.
- We design and implement an RPN (region proposal network)-FPN that combines multi-scale voxel features efficiently and succinctly in a later fusion manner.
- Our system runs at 50 FPS and achieves good detection results according to BEV and 3D detection.

The organization of this paper is as follows. Section 2 reviews some related literature. The details of the proposed Voxel-FPN method are introduced in Section 3. Some experiments are given in Section 4. Section 5 discusses the proposed method in more depth. Section 6 concludes this paper.

## 2. Related Work

This section briefly reviews some point cloud-based 3D object detection methods.

### 2.1. Point Cloud Representation Learning Methods

Recent years have witnessed major breakthroughs in point-cloud processing literature. Many point cloud processing methods are hand-crafted features towards specific tasks. Point cloud features usually encode certain statistical properties of points and are designed to be invariant to certain transformations. These features are usually designed to preserve the inherent or external properties of point clouds and handle the invariance of point clouds. These methods can be divided into two categories based on the retained information. The works in [21,22,23] retain local features of the point cloud, whereas the works in [24,25,26,27,28] focus on global features of the point cloud. For a specific task, it is not easy to find the best combination of features.

Recently, a method called PointNet [29] that can automatically learn features from point cloud has emerged. PointNet applies several nonlinear transformations and max-pooling operation on point clouds to predict classification distribution. Due to the shared transforms for each point, the size of learnable parameters is small in practice and the time consumption is relatively low. Moreover, with fully connected layers only affecting on feature dimension, PointNet is irrelevant to the input size of the point sets, allowing point clouds of variable input sizes as input.

When treating each point with shared mapping and exploring global information by observing correlation of point sets entirely, PointNet may omit local features at different local scales. Therefore, PointNet++ [30] focuses on building rich hierarchical features from neighboring points. In [30], a set abstraction layer consists of sampling local centers, grouping and applying a standard PointNet module to aggregate features of local regions. Set abstraction layers aim to extract features hierarchically and then upsampling operation with interpolation followed by unit PointNet makes output features pointwise.

The advantage of utilizing PointNet is the great flexibility of producing pointwise or global features without requiring any prior knowledge of topology. Along with learned features, points could be further voxelized or grouped as well as the original ones. Such a merit makes PointNet a fundamental component that could be easily embedded in 3D detection frameworks. For example, voxel feature encoding (VFE), proposed in [31], groups points into voxels based on their local coordinates. Similar to PointNet’s architecture, linear transforms and max-pooling are conducted in each voxel cells to aggregate features. Voxel-wise features are then collected and transported into middle layers to form descriptive information on the shape of local neighborhood.

### 2.2. Fusion-Based 3D Object Detection

In the field of 3D object detection, some methods fuse point clouds and images to achieve the goal.

MV3D [7] and AVOD [8] project point cloud into multiple views, e.g., in BEV (Bird’s Eye View) or front view, stack features from corresponding regions in RGB images and finally fuse them to generate a comprehensive representation for detection. Due to misalignment between 2D images and sparsely distributed points in 3D space, a bottleneck may exist in such fusion-based methods. Nevertheless, combined efforts should be made to efficiently capture and unify a coupled representation for various data modalities.

F-PointNets [10] extends the 2D detection results into corresponding frustums in the 3D space. Afterwards, it employs a PointNet/PointNet++ to segment points into binary (foreground or background) classes and then makes a regression using foreground points. The assumption that only one object exists in a search region is crucial but somehow contradicts with occasions where several objects are closely located and occluded in front view, causing more than one objects in a single frustum. On the other hand, such a detection method relies largely on the quality of the input 2D proposals.

### 2.3. Point Cloud-Based 3D Object Detection

In recent years, more and more attention has been paid to 3D object detection based on pure point clouds. These methods can be roughly categorized as BEV-based, point-based, and voxel-based.

BEV-based methods first transform the point cloud data into BEV representation through some rules, and then use learning methods to learn and predict the target. ComplexYOLO [32] converts the point cloud to BEV form, uses the expanded form of YOLO [4] as the backbone network, and then uses a complex regression strategy to estimate multi-class 3D boxes. PIXOR [33] uses a specific height encoding method to represent the point cloud as BEV, and then designs a single-stage proposal-free dense object detector for 3D object detection. However, a key problem with these BEV-based methods is that when generating BEV maps, many data points are discarded, resulting in a considerable information loss. This loss of information impairs the performance of these methods for 3D object detection.

The point-based method is a recently emerging method, of which the representative is PointRCNN [13]. PointRCNN is a two-stage 3D detector. It first extracts pointwise features and regards each point as a regression center for candidate proposals. To reduce overwhelming number of input points, PointRCNN uses standard PointNet++ to segment points in the first stage and only treats foreground ones as regression targets. In the second stage, generated 3D proposals are then gathered with locally aggregated features in region of interest (ROI) for localization and classification refinement. Usually, the detection accuracy of PointRCNN is better than the other two categories, but its detection speed is very low. Therefore, improving detection speed while ensuring detection accuracy is a good direction for PointRCNN in the future.

The voxel-based method is one of the currently popular 3D detection methods. The authors of [11,16] use six statistical quantities to encode nonempty voxel, and then a novel convolution-like method is applied to detection. However, such approaches may be suboptimal, as handcrafted feature extraction methods may not be generalizable to new environments without much engineering effort. To address this problem, VoxelNet [31] uses simplified PointNet to automatically learn features of each voxel, and for the first time, an end-to-end learning method is implemented in the voxel-based domain. It groups points into voxels, extracts features in each voxel by applying a PointNET and aggregates the extracted voxel-wise features in middle convolutional layers for detection. On the observation of sparsity in non-empty voxels, SECOND [12] designs sparse convolution algorithm and integrates it to the original framework of VoxelNet to accelerate the calculation of convolutional layers. With the prior knowledge of no overlapping between two objects in height dimension, PointPillars [19] further simplifies SECOND by implementing voxelization only in the BEV plane. As named, points are grouped into vertical columns instead of strided voxels. In comparison with heavy computations of 3D convolution in VoxelNet, PointPillars shifts to 2D convolution, thus greatly reducing the space and time complexity of point feature extraction. Similar to the voxel-based method, our method integrates multi-voxel segmentation sizes to improve the detection accuracy and ensure the real-time detection.

## 3. Voxel-FPN Detector

In this section, we introduce the structure of the proposed Voxel-FPN method and details of each part of the algorithm. Implementation details of the proposed method are also introduced.

### 3.1. Motivation

To directly use the powerful CNN for training and inference on the point cloud, voxelization of the point cloud is a very popular method [19]. However, the single-dimensional voxelization of the point cloud will lose some of the local structure of the point cloud, which will result in the inability to learn rich feature representations [19]. Multi-scale voxel segmentation can alleviate the above problem and retain more local structures, but the effective combination of multi-scale features has great challenges.

To this end, the proposed method is new to exploit multi-scale voxels and feature pyramid network. The two Effective modules are Voxel Feature Encoding (VFE)-FPN to obtain and fuse voxelization representations in different sizes, and region proposal network (RPN)-FPN to efficiently fuse multi-scale voxelization features. The proposed method is elaborated on in the following.

### 3.2. Network Architecture

**Figure 1:**
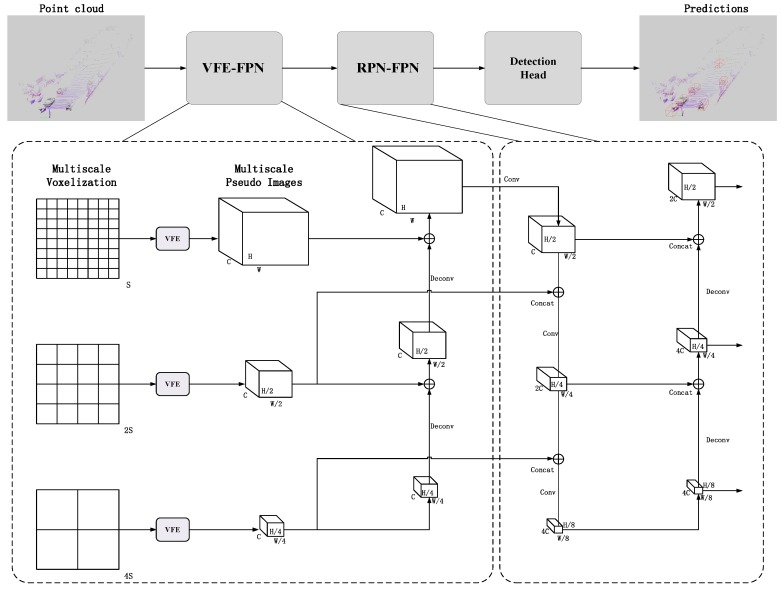
The structure of the network. The detector takes the raw point cloud as input, converts it into multiple-scale voxels, and uses voxel feature encoding VFE-FPN to extract and fuse voxel features of different sizes. Then, an RPN-FPN network is used to integrate multi-scale features. Finally, detection head (Single Shot Detector (SSD)) is used for prediction.

The pipeline of the proposed framework, shown in Figure 1, consists of two major blocks: VFE-FPN, which divides raw point clouds into voxels of multiple scales, uses a simple PointNet to learn voxel features, and then a simplified FPN network is designed to fuse voxel features of different sizes, and multi-scale features aggregation, in which a region proposal network with feature pyramid structure is designed to achieve the function of integrating multi-scale original features, and output the detection results (noted as RPN-FPN).

### 3.3. Multi-Scale Voxel Features Learning (VFE-RPN)

#### 3.3.1. Multi-Scale Voxelization

Considering point cloud’s uneven density across the 3D space, a single setting for default voxel grids can be insufficient to represent all the information available in the scenario. Consequently, we propose a densely aggregated structure to encode voxel feature from multi-scales in a bottom-up way.

The basic voxel partition method is as follows. The 3D space, ranging from W,H,D along the X,Y,Z axes, are divided into equally distributed voxel cells, denoted as ($v_{W},v_{H},v_{D}$), thus producing voxel grids of size ($W/v_{W},H/v_{H},D/v_{D}$). Points are then grouped into those voxels based on individual coordinates (x,y,z). Due to the sparsity of point clouds, point number in each voxels may vary. To reduce learning bias, points are randomly sampled with the same number *N* for each voxel: If a voxel holds too much data, the data is randomly sampled with a fixed number *N*; conversely, if a voxel has too little data, zero padding is applied.

Using the above voxel partition method and changing the size of a single voxel, a multidimensional voxelization input can be obtained. Noting a base voxel size as S, we iteratively voxelize a 3D space into multiple sizes of S, 2S, 4S etc., which is given in a serial size of S multiplied by powers of 2. Driven by various combinations of points as input, voxels in multiple scales produce various features.

#### 3.3.2. Multi-Scale Features Learning

**Figure 2:**
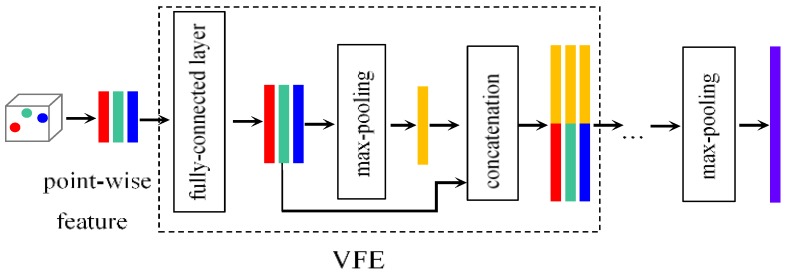
Structure of voxel feature extraction network.

Points in each voxel are collected to form the input feature. Then Voxel Feature Encoding (VFE) [31] blocks, shown in Figure 2, are utilized to extract voxel-wise features. A VFE block consists of a fully-connected layer, max-pooling and pointwise concatenation to learn pointwise features. After several VFE blocks applied, an element-wise max-pooling operation is conducted to obtain an aggregated output. As a result, input point cloud is transformed into voxel-wise features. By inputting voxelization of each size to the VFE module, pseudo image features of different sizes can be obtained.

#### 3.3.3. Multi-Scale Features Early Fusion

This study uses a multiple of 2 to continuously enlarge the size of the voxel, so the height and width of the obtained pseudo image features are continuously reduced by a factor of 2. Assuming the width and height of pseudo image features obtained by the size S are (H,W), the width and height of pseudo image features obtained by 2S and 4S are (H/2,W/2) and (H/4,W/4). To fuse the features of each scale, two consecutive upsamplings are performed in this paper, and small-scale feature is gradually merged into large-scale feature maps. The fused features are passed into the subsequent RPN-FPN network.

The advantage of this early fusion method is that the features learned through VFE are elementary point cloud features, and fusion of these features in the early stage can make subsequent CNN networks summarize more useful information.

### 3.4. Multi-Scale Voxel Features Later Fusion

Efficiently merging features of different scales is a huge challenge. In the automatic driving task, 3D detection requires high real-time performance; otherwise, it will have serious consequences. Many of the current integration schemes are very complex, such as MV3D [7] and AVOD [8]. These solutions will bring loss of detection time. To use multi-scale features simply and efficiently, this paper designs an RPN-FPN network, which can easily make full use of multi-scale features and achieve good detection results.

As one of the basic components in modern detectors, the region proposal network serves as key module to decode the input feature maps and transform them to candidate boxes. In this work, with the feature maps generated from various voxels of multiple scales as the input to RPN, we adopt Feature Pyramid Network (FPN) [20] design to combine multi-scale features of point cloud. Accordingly, we feed feature maps from high resolution into multiple convolutional block with a scaling step of 2. Then, downsampled outputs are concatenated with voxel feature from coarsen resolution and merged as the input for next scale, thus forming a hierarchy of aggregated features across various voxel scales. Besides the bottom-up path used in multi-scale feature aggregation, we build a top-down FPN to efficiently construct a rich, hierarchical feature pyramid for multiple voxels features. Each level of the voxel pyramids can be utilized to detect 3D objects in the corresponding regions.

The right of Figure 1 shows the structure of the RPN-FPN block. With coarser input, we upsample the resolution in x-y plane by a factor of 2. The upsampled map is then merged with the corresponding map by a concatenation operation, where the corresponding map refers to the convolution map of the corresponding size in the downsampling process of RPN-FPN block. For example, a map of (H/4,W/4) obtained by upsampling (H/8,W/8) is connected to a map of (H/4,W/4) obtained in the convolutional network. We iterate this process until the finest resolution is met. The final set of voxel features is denoted $P_{1},P_{2},P_{3}$, corresponding to $C_{1},C_{2},C_{3}$ that are, respectively, of different voxel sizes and ranges. Detection heads are attached on feature pyramid and each default 3D anchors are assigned with corresponding maps, outputting class prediction, location regression, and orientation prediction.

This relatively later fusion method can preserve the rough features obtained by large voxel division to the greatest extent, and this feature will have a good effect on long-range object detection. This is also verified in subsequent experiments.

### 3.5. Fusion Strategy

This study provides two fusion strategies, which are divided into the following versions according to the stage of feature fusion:

**Voxel-FPN V1:** Only the early feature fusion method is used, and the later fusion method is not applied. That is, the primary multi-scale voxel features will not be directly fused with the RPN-FPN network, but will be upsampled and re-fused through the VFE-FPN network.

**Voxel-FPN V2:** Only the later fusion method is used, and the early fusion method is not applied. Under this strategy, multi-scale voxel features are not upsampled by the VFE-FPN network, but are directly merged with the RPN-FPN network.

**Voxel-FPN:** Both fusion methods are used to take full advantage of the advantages of the two fusion methods.

### 3.6. Implementation Details

#### 3.6.1. Network Details

We use conv(cout,k,s) to represent a standard 2D convolutional block, where cout, *k*, and *s* denote the output channel number, kernel size, and stride, respectively. Three convolution blocks are used in RPN, among which block-1 consists of three convolution layers (conv(64,3,2)−conv(64,3,1)−conv(64,3,1)), then five convolution layers (conv(128,3,2)−conv(128,3,1)−conv(128,3,1)−conv(128,3,1)−conv(128,3,1)), and then five convolution layers (conv(256,3,2)−conv(256,3,1)−conv(256,3,1)−conv(256,3,1)−conv(256,3,1)) are applied to block-2 and block-3, correspondingly. The other two voxel feature maps are concatenated to the corresponding feature maps in the convolutional network with the same shapes.

**Figure 3:**
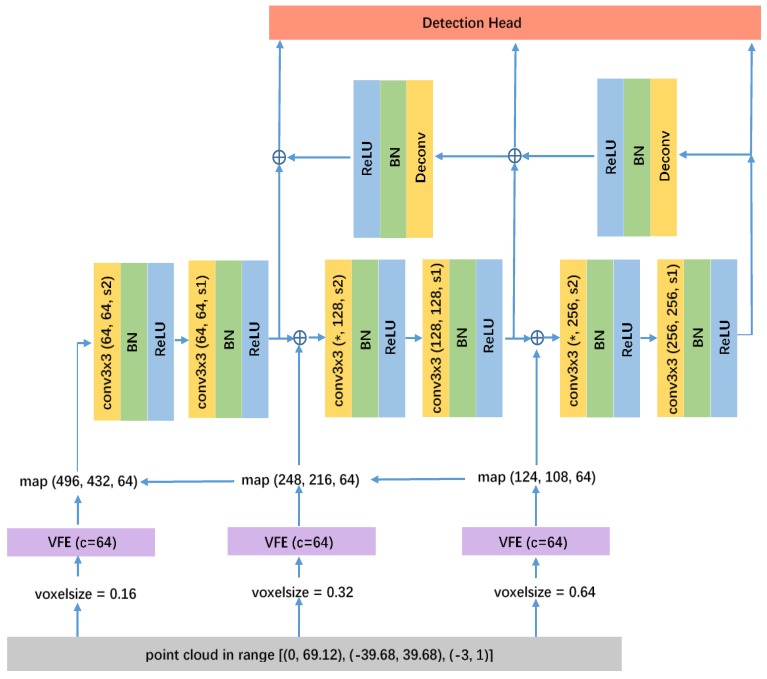
The detailed structure for RPN-FPN.

The detailed implementation of Feature pyramid network in RPN is illustrated in Figure 3, in which “*” represents number of output channels depending on the merging strategy among intermediate layers. We compare two forms of merging operations: “add” or “concat”. Network utilizing concat operations has better performance than that using add, while other configurations are kept the same. In convolutional layers of Figure 3, $conv\;k\times k(n_{1},n_{2},s)$ denotes a convolutional module with kernel size k×k, input channels $n_{1}$, output channels $n_{2}$ and stride *s*. As there are only three convolutional layers in the backbone of network, two additional feature maps are produced from VFE before multi-scale voxel feature aggregation. However, in more common cases, design of multi-scale voxel feature aggregation may require more feature maps of different sizes from VFE to be considered, as the same number with convolutional layers in the backbone. Three feature maps are drawn in the Figure 3 for illustration. BN denotes a batch norm layer in Figure 3. Upsample layers are implemented by deconvolutional modules, which are denoted as Deconv in Figure 3. After downsampled by convolutional layers, features are unsampled by deconvolutional layers, which parameters are (256,128,2,2) and (256,64,2,2), respectively. Finally, detection headers are applied after the last convolution of each block sequentially.

#### 3.6.2. Anchors and Targets

In a way similar to PointPillars method, with prior knowledge of no overlapping cars in height, the range of x,y,z in point cloud is restricted to [(0, 69.12), (−39.68, 39.68), (−3, 1)]. The car anchor has width, length, and height of (1.6, 3.9, 1.5) m with a *z* center of −1 m. Accordingly, three resolution in x−y plane is used to voxelize the point cloud space, producing voxel size of S, 2S, 4S each. Unless otherwise noticed, the implementation settings below is based on S = 0.16 m. Here, the selection of 0.16 m is selected by the validation set detection performance. When readers apply the proposed method to their own application scenarios, they can first set multiple sizes, such as 0.16 m, 0.20 m, 0.24 m, etc., and then select the best size based on the detection performance of the validation set. The max number of points per voxel is set to [100, 200, 300] and that of voxels is [12000, 8000, 6000]. Then two layers of VFE with 64 output channels are applied in points for voxel feature extraction. After voxelization, point feature ($x_{i},y_{i},z_{i},r_{i}$) in each voxel is concatenated with their offset to average point coordinates ($x_{i}-\bar{x},y_{i}-\bar{y},z_{i}-\bar{z},x_{i}-\bar{x_{p}},y_{i}-\bar{y_{p}}$) as VFE input, denoted as ($x_{i},y_{i},z_{i},r_{i},x_{i}-\bar{x},y_{i}-\bar{y},z_{i}-\bar{z},x_{i}-\bar{x_{p}},y_{i}-\bar{y_{p}}$), where $r_{i}$ is reflectance. Therefore, a voxel with point feature of seven channels is transported into VFE to produce voxel feature of 64 channels. By doing this, we obtain three different voxel feature maps with tensor shapes of (432, 496, 64), (216, 248, 64), and (108, 124, 64), then the largest feature map with a tensor shape (432, 496, 64) becomes the base part of a convolutional network.

Similar to other voxel-based methods in literature, the proposed 3D object detection network utilizes the same localization loss function proposed in [12]. For regression task, the residual targets can be encoded with the following equations,

(1) $$\begin{array}{cc} x_{t} & =\frac{x^{g}-x^{a}}{d^{a}},y_{t}=\frac{y^{g}-y^{a}}{d^{a}},z_{t}=\frac{z^{g}-z^{a}}{h^{a}},\\ w_{t} & =\log\frac{w^{g}}{w^{a}},l_{t}=\log\frac{l^{g}}{l^{a}},h_{t}=\log\frac{h^{g}}{h^{a}},\\ \theta_{t} & =\sin\left(\theta^{g}-\theta^{a}\right). \end{array}$$

where x,y,z are the center coordinates of the 3D bounding box; w,l,h are the width, length, and height, respectively; θ is the heading angle; $d^{a}=\sqrt{{\left(l^{a}\right)}^{2}+{\left(w^{a}\right)}^{2}}$; and subscripts *t*, *a*, and *g* indicate the encoded regression targets, the anchor, and the labeled bounding box, respectively.

#### 3.6.3. Loss

There are two types of loss: the regression loss of the position and the classification loss. Total localization loss is given by

(2) $$\begin{array}{cc} L_{\mathrm{loc}} & =\sum_{res\in \left(x,y,z,w,l,h,\theta \right)}\mathrm{SmoothL}1(\Delta res),\\ \mathrm{SmoothL}1(x) & =\left\{\begin{array}{cc} 0.5x^{2}, & \;if|x|<1,\\ |x|-0.5, & \;otherwise. \end{array}\right. \end{array}$$

For classification branch of the detection output, focal loss is used to handle the imbalance of positive and negative samples:

(3) $$L_{\mathrm{cls}}=-\alpha_{a}{\left(1-p_{a}\right)}^{\gamma }\log\left(p_{a}\right).$$

The regression scheme mentioned above treats boxes with opposite direction as being the same, so a direction classifier is added to the output of RPN. $L_{\mathrm{dir}}$ term is a softmax classification loss on the discretized directions as in [13]. Altogether, the overall loss is as follows,

(4) $$L_{\mathrm{total}}=\beta_{0}L_{\mathrm{cls}}+\frac{1}{N_{pos}}\left(\beta_{1}L_{\mathrm{loc}}+\beta_{2}L_{\mathrm{dir}}\right).$$

where $\beta_{0}=1.0,\;\beta_{1}=2.0,$ and $\;\beta_{1}=0.2$, and $N_{pos}$ is the number of positive anchors.

#### 3.6.4. Data Augmentation and Opimization

In terms of data augmentation, global flipping, rotation and scaling is applied to the whole point cloud scenarios, where the flipping probability is set to 0.5, the rotation angle ranges from [−π/4,π/4], and the scaling ratio is between [0.95, 1.05]. Followed by [13], during the training process of detection network, several new ground truth boxes and the corresponding points will be put to the same locations of the current scene. Physical collision is tested to ensure only nonoverlapping boxes will exist. The object detection network is trained for 160 epochs with Adam optimizer, the initial learning rate is 0.0002, and with decay factor 0.8 and a decay period every 15 epochs.

## 4. Experiments

In this section, we focus on extensive experiments and analysis to optimize the network structure and verify the effectiveness of our framework on 3D car detection task.

### 4.1. Dataset

All experiment are conducted on KITTI dataset [34], which contains 7481 training and 7518 test pairs of both RGB images and point cloud data. As the ground-truth annotation are held for benchmark only, we further split the original dataset into 3712 training and 3769 validation samples to verify the effectiveness of the proposed framework [35]. As objects visible in the image are annotated, we retain point clouds that can be projected to the front camera, which roughly contain 20k points for each scenario. Our computation environment for inference is an Intel i7 CPU and a 1080Ti GPU.

### 4.2. Evaluation Using the KITTI Test Set

**Figure 4:**
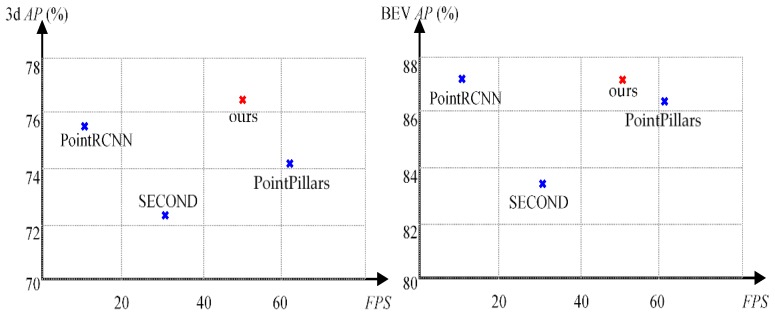
AP metric of car 3d and BEV detection in moderate level compared with some top-ranking approaches.

Shown in Table 1, we compare performance of our Voxel-FPN method with other approaches in the public KITTI 3D object detection benchmark. Among these approaches, some top-ranking ones in 3D detection and BEV are selected out and their APs (average precision) in moderate level are drawn in Figure 4 to illustrate the tradeoff between comprehensive performance and time consumption. In AP metric, our method has achieved an excellent performance, especially in moderate level of 3D detection, which is an important metric for autonomous driving. In comparison with two other voxel-based approaches, i.e., SECOND and PointPillars, our method has better performance. We attribute this to feature representation enhanced by multi-scale voxel feature aggregation.

As reported in work [20], the FPN pathway only introduces relatively small burden due to additional convolutional layers. As seen in Figure 4, blessed with point cloud data input only, the proposed method has less running time compared to most of the top ranking methods. The only faster method among them, i.e., PointPillars, has lower AP than ours. We believe that the simplicity and efficiency of Voxel-FPN will benefit other point cloud based 3D detectors and serve as a better feature extraction module in future fusion based systems.

**Figure 5:**
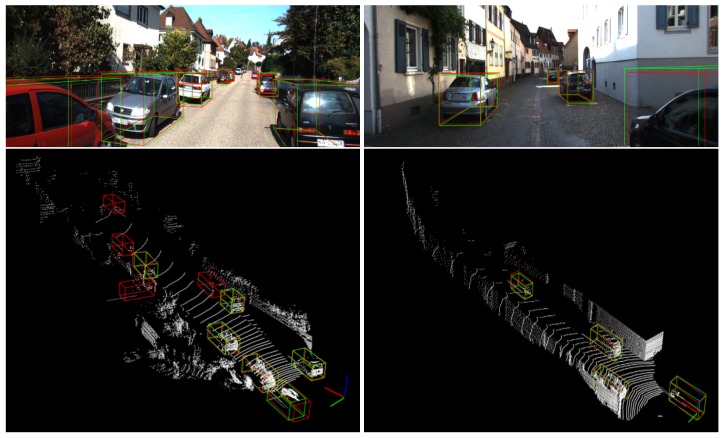
Visualized car detection results from our method: cubes in green color denote groundtruth 3D boxes and those in red indicate detection results.

Figure 5 shows visualized outputs of our proposed framework. We illustrate both projected boxes on RGB image and 3D detection results on point cloud data. In the left column of Figure 5, a car behind a tree and a white car could be detected out, which is severely occluded and removed from groundtruth annotations in dataset. In the right column of Figure 5, the car near right boundary is successfully detected, showing the capacity of capturing truncated objects in front eye view.

**Figure 6:**
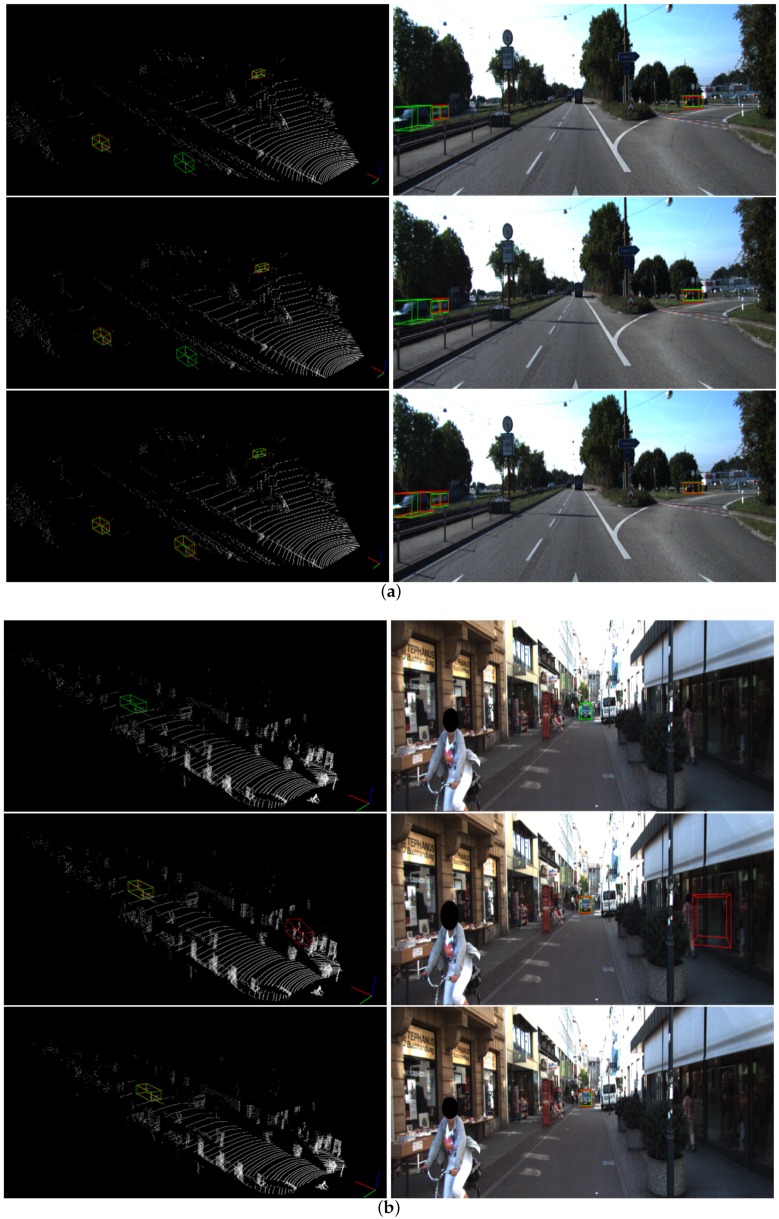
Comparison of results from SECOND (top), PointPillars (middle), and ours (bottom) for two diferent scenes (**a**) and (**b**).

In terms of voxel-based methods, detection results from SECOND, PointPillars and ours are visualized in Figure 6 for a fair comparison. Under the observation of scenes in validation set, we can inspect that SECOND is more likely to miss objects while PointPillars tends to have more false detections, e.g., in Figure 6b. In comparison, results from Voxel-FPN outperforms these two approaches and we attribute this mainly to the enhanced feature presentation by multi-scale voxel feature aggregation.

### 4.3. Evaluation Using the KITTI Validation Set

The results of the KITTI validation set are shown in Table 2. It can be seen from the table that the proposed Voxel-FPN method is superior to the comparative methods in all levels of difficulty, and has a fast inference speed.

### 4.4. Ablation Studies

Extensive ablation experiments are conducted to probe the contribution of each component.

#### 4.4.1. Pedestrian and Cyclist detection

**Figure 7:**
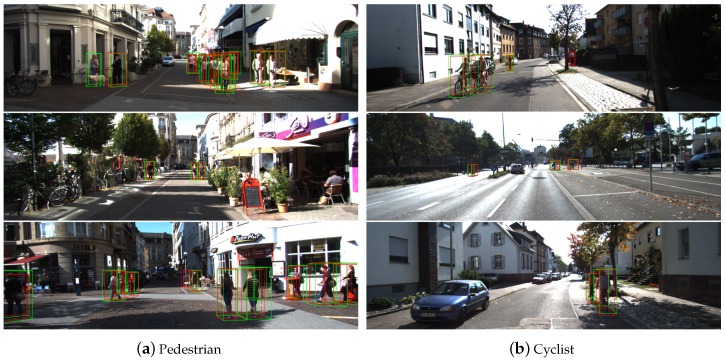
Results of 3D detection of Pedestrian and Cyclist on the KITTI validation set.

In this section, the present study shows the results of the proposed method for the detection of pedestrians and cyclists. The range of x,y,z in point cloud for pedestrians and cyclists is [(0, 48), (−20, 20), (−2.5, 0.5)] m, respectively. The pedestrian anchor has width, length, and height of (0.6, 0.8, 1.73) m with a *z* center of −0.6 m, whereas the cyclist anchor has width, length, and height of (0.6, 1.76, 1.73) m with a *z* center of −0.6 m. No published method shows the results of pedestrians and cyclists in the KITTI validation set except for VoxelNet [31]. Therefore, this section only provides comparison results with VoxelNet method. The proposed method is also an improvement of the voxelization method, so comparison with VoxelNet is also meaningful. It can be seen from Table 3 that the proposed method has better detection performance for pedestrians and cyclists than VoxelNet. Figure 7 also shows some detection results. The above results show that the proposed method can detect pedestrians and cyclists to a certain extent. However, note that the detection rate is not high enough. This is because, first, pedestrians and cyclists are much smaller than cars, resulting fewer points per instance, and second, the dataset contains very few pedestrians and cyclists objects (2207 and 734 respectively), which makes the model difficult to train. Studying effective models for detecting pedestrians and cyclists is one of the key points of future research.

#### 4.4.2. Different Ranges Performances

To verify the detection performances of voxel partition of different sizes for objects with different distances, this paper gives three ranges of detection results: 0–30 m, 30–50 m and >50 m. The evaluation results are listed in Table 4.

It can be seen that the larger the size, the better the detection performance for farther objects. This shows that the local features retained by voxels of different sizes are complementary. At the same time, the table also shows that the detection performance of Voxel-FPN v2 is better than V1 at long range, which indicates that the later fusion method retains the original features to a greater extent.

#### 4.4.3. Different Fusion Strategies Performances

Table 5 reports the results of different fusion strategies on the validation set. It can be seen that V1 and V2 have different effects on different detection standards and difficulty levels, which shows that the two fusion methods can deal with different problems. Therefore, the combination of the two methods is superior to the method using only one fusion method in all aspects. However, the model complexity of the combination method is higher, so appropriate fusion method can be selected for practical problems.

#### 4.4.4. Detection with the Actual Equipment in the Actual Operation Scenario

**Figure 8:**
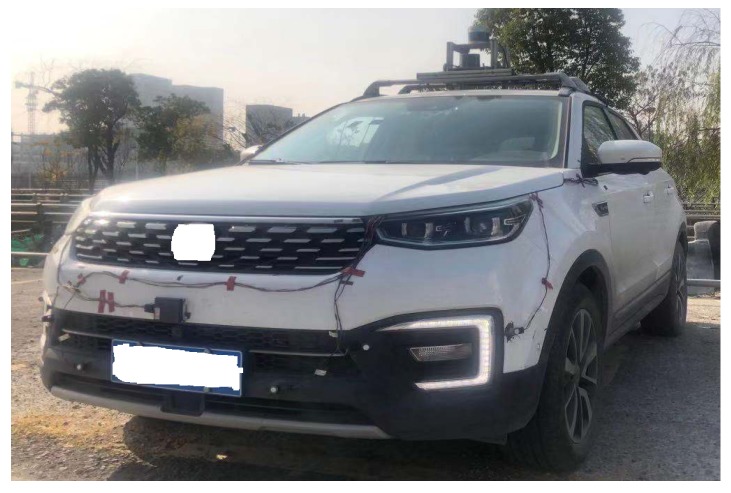
Test vehicle configuration.

**Figure 9:**
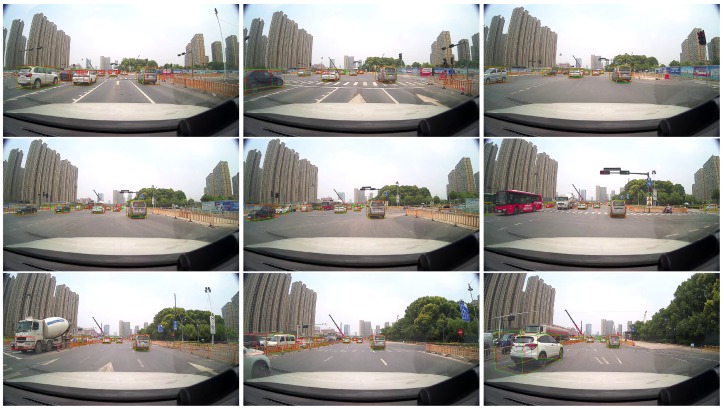
Results of 3D detection in actual scenario. The above are scenes of 9 consecutive frames, and the order is from left to right and then from top to bottom.

This section shows the application of the proposed algorithm in actual scenarios. Figure 8 shows an autonomous vehicle used in actual scenario, with a LIDAR device on the roof. The vehicle has collected data for actual road scenarios. Figure 9 shows the detection results of the actual scene by the proposed algorithm, and it are continuous multi-frame scenes from top to bottom. It can be seen that the proposed algorithm can effectively detect objects, and objects between frames are continuous.

## 5. Discussion

This section further discusses the proposed method.

It can be found from the experimental results that the proposed method cannot currently output multiple classes at the same time, for example, it can only predict car or non-car, or only pedestrians and cyclists. This is because the size difference between car and pedestrians and cyclists in reality, and the Anchor generation method of the proposed method is divided by the actual size, which will lead to a decrease in accuracy when predicting multiple classes at the same time. Therefore, researching effective 3D detection methods that can predict multiple types at the same time is the future research direction.

The proposed method currently has low prediction accuracy for pedestrians and cyclists. Pedestrians and cyclists have relatively few points in the point cloud. When the distance is farther, the point cloud is almost absent, which leads to a significant reduction in the performance of pure point cloud algorithms. In the future, we can consider the use of image information to design algorithms for image and point cloud fusion to overcome the above problem.

## 6. Conclusions

At present, voxel-based 3D object detection methods only use a single-scale voxel partition method, and thus some local spatial information contained in original point clouds will be lost. In this paper, we propose Voxel-FPN, a novel one-stage end-to-end trainable deep architecture for multi-scale voxel partitions. We have designed two fusion methods to effectively fuse multi-scale voxel features while ensuring the real-time performance of the calculation. Our approach encodes multiple scales of voxel grids from different reception fields and decodes them to final feature maps via a top-down pyramid network, forming a rich hierarchy of feature maps. Experimental studies indicates that our method is not only competitive in 3D detection outputs, but also save great time complexity to be applied in real-world inference tasks. However, our network does not consider image information. Future work will focus on the exploration of fusion methods of images and point clouds to enhance detection performance.

## Figures and Tables

**Table 1:** 3D detection and Bird’s eye view detection performance of car (IoU(Intersection over Union) = 0.7): Average Precision (AP) (%) in the KITTI test set.

Method	3D detection			BEV			Modality	Scheme	FPS
	Easy	Mod	Hard	Easy	Mod	Hard			
FPointNet	82.19	69.79	60.59	91.17	84.67	74.77	LIDAR + RGB	2-stage	6
MV3D	74.97	63.63	54.00	86.62	78.93	69.80	LIDAR + RGB	2-stage	3
AVOD	83.07	71.76	65.73	90.99	84.82	79.62	LIDAR + RGB	2-stage	10
PointRCNN	86.96	75.64	70.70	92.13	87.39	82.72	LIDAR	2-stage	10
SECOND	83.34	72.55	65.82	89.39	83.77	78.59	LIDAR	1-stage	25
PointPillars	82.58	74.31	68.99	90.07	86.56	82.81	LIDAR	1-stage	62
Voxel-FPN	85.64	76.70	69.44	92.75	87.21	79.82	LIDAR	1-stage	50

**Table 2:** 3D detection and Bird’s eye view detection performance (AP) (%) of car in the KITTI validation set.

Method	3D detection			BEV			FPS
	Easy	Mod	Hard	Easy	Mod	Hard	
F-PointNet	83.76	70.92	63.65	88.16	84.02	76.44	6
MV3D	71.29	62.68	56.56	86.55	78.10	76.67	3
VoxelNet	81.97	65.46	62.85	89.6	84.81	78.57	4
SECOND	87.43	76.48	69.10	89.96	87.07	79.66	25
PointPillars	86.13	77.03	72.43	89.93	86.92	84.97	62
Voxel-FPN	88.27	77.86	75.84	90.2	87.92	86.27	50

**Table 3:** 3D detection performances of pedestrians and cyclists (IoU = 0.5).

Method	Pedestrian			Cyclist		
	Easy	Mod	Hard	Easy	Mod	Hard
VoxelNet	57.86	53.42	48.87	67.17	47.65	45.11
Voxel-FPN	85.25	64.36	61.00	68.77	61.86	56.40

**Table 4:** Detection performances of partition methods with different voxel sizes in different ranges (S = 0.16 m).

Method	easy			mod			hard		
	0–30 m	30–50 m	50 m-inf	0–30 m	30–50 m	50 m-inf	0–30 m	30–50 m	50 m-inf
S	87.94	42.47	-	88.54	52.89	5.24	81.63	51.01	5.76
2S	88.61	45.38	-	88.83	51.29	7.62	84.55	48.43	7.83
4S	84.08	31.61	-	86.35	42.05	10.49	78.04	40.38	9.75
Voxel-FPN V1	88.56	48.27	-	88.89	54.11	10.97	85.71	52.15	11.21
Voxel-FPN V2	88.47	47.9	-	88.91	53.6	13.16	85.4	51.74	13.21

**Table 5:** Detection performances of different fusion strategies.

Method	3D detection			BEV		
	Easy	Mod	Hard	Easy	Mod	Hard
Voxel-FPN V1	87.43	77.68	75.04	90.09	87.93	86.17
Voxel-FPN V2	87.36	77.72	74.85	90.13	87.79	86.02
Voxel-FPN	88.27	77.86	75.84	90.2	87.92	86.27

## Abbreviations

The following abbreviations are used in this manuscript:

FPN	Feature Pyramid Network
RPN	Region Proposal Network
VFE	Voxel Feature Encoding
